# Liquid-Phase Exfoliated 2D Nanomaterials for Enhanced Vibrational Circular Dichroism of Chiral Molecules

**DOI:** 10.1021/acs.nanolett.5c03583

**Published:** 2025-09-17

**Authors:** Aria T. Ballance, Urice N. Tohgha, Amy Morren, Michael A. Susner, Jennifer S. Shumaker-Parry, Peter R. Stevenson

**Affiliations:** † Department of Chemistry, 7060 University of Utah, Salt Lake City, Utah 84112, United States; ‡ Air Force Research Laboratory, Materials and Manufacturing Directorate, Wright-Patterson Air Force Base, Dayton, Ohio 45324, United States; § Azimuth Corporation, a Core4ce LLC, 2970 Presidential Drive #200, Fairborn, Ohio 45342, United States

**Keywords:** 2D Materials, Exfoliation, MXene, Thin Film, TMDC, Vibrational Circular Dichroism

## Abstract

Vibrational circular dichroism (VCD) spectroscopy is a powerful technique for stereochemical analysis, but its application is often limited by inherently weak polarization-dependent signal absorption intensities (e.g., 10^–4^ to 10^–5^). This study investigates liquid-phase exfoliated (LPE) two-dimensional (2D) nanomaterials as a readily accessible and versatile platform for enhancing molecular VCD signals. We explored the optical interactions between chiral molecules (R)- and (S)-1,1’-bi­(2-naphthol) (BINOL) and a range of LPE 2D materials possessing diverse electronic properties: semiconducting 2H-WS_2_, semimetallic 2H-TiS_2_ and 2H-NbS_2_, metallic 3R-NbS_2_ and Ti_3_C_2_T_*z*_, and magnetic metallic 2H-VS_2_. Significant VCD signal enhancement for BINOL was observed, reaching up to 2 orders of magnitude relative to BINOL alone. Overall, the 2D materials induced varied effects, including signal enhancement, signal dampening, peak shifts, and inversion of VCD handedness. This work demonstrates potential platforms for modulating and enhancing molecular VCD characteristics, offering a promising route toward more sensitive chiroptical molecular analysis and detection.

## Graphical abstract

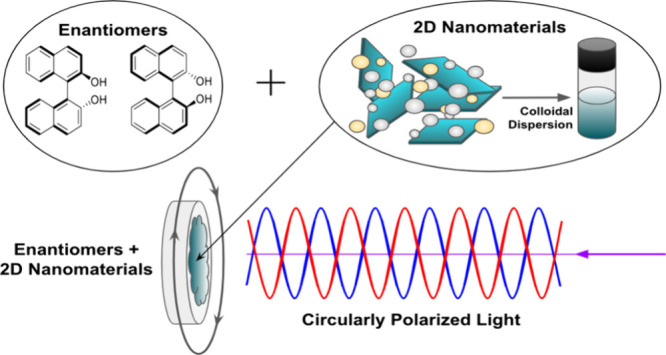

Vibrational circular dichroism (VCD) spectroscopy, the differential absorption (A) of left (L) and right (R) circularly polarized infrared light (ΔA = A_L_ – A_R_), provides rich structural information about chiral molecules in solution and the solid state.^1–5^ It is particularly valuable for determining absolute configuration in conjunction with density functional theory (DFT) calculations and for studying conformational equilibria and supramolecular chirality in complex systems like biomolecules.^1,6,7^ However, the inherent weakness of VCD signals (typically 4 to 5 orders of magnitude lower than the intensity of the parent infrared, IR, absorption band) often necessitates high analyte concentrations or prolonged acquisition times, limiting its applicability, especially for trace analysis or weak optically absorbing molecular species.^5,8^

To overcome this sensitivity limit, significant effort has been focused on developing surface-enhanced chiroptical spectroscopic techniques. For example, in relation to VCD, surface-enhanced Raman scattering (SERS) is well-established,^9–11^ and surface-enhanced infrared absorption (SEIRA) or surface-enhanced vibrational spectroscopy (SEVS) has also gained prominence.^8,12–15^ Enhancement platforms for such techniques typically rely on resonant interactions between molecules and plasmonic nanostructures, usually fabricated from noble metals like gold or silver via lithographic or chemical synthesis methods.^16–18^ While functional, these plasmonic platform approaches often involve complex, costly, and defect-induced nano- or microfabrication. Additionally, in some cases, the strong optical response of the plasmonic material itself can interfere with the interpretation of the surface-enhanced signal.^19–22^ Furthermore, to achieve ideal surface-enhanced behavior, chiral plasmonic structures designed for surface-enhanced spectroscopy often require sophisticated AI/ML computational optimization and multistep experimental fabrication.^23–25^ Such considerations motivate the exploration of alternative nanomaterial systems and simpler strategies for molecular spectroscopic signal enhancement.

Two-dimensional (2D) nanomaterials, such as transition metal dichalcogenides (TMDCs) and transition metal carbides or nitrides (MXenes), represent compelling alternative materials classes for spectroscopic molecular sensing applications. These crystalline materials consist of atomically thin layers held together by van der Waals forces, thus allowing for delamination from bulk precursors into few-layer nanosheets using methods like liquid-phase exfoliation.^26–28^ Liquid-phase exfoliation, frequently aided by sonication in suitable solvents or surfactant solutions, offers a scalable, cost-effective, and versatile route to producing 2D material dispersions compared to intricate nanofabrication techniques. The resulting nanosheets exhibit exceptionally high surface-to-volume ratios, providing an abundant surface area for interactions with analyte molecules. Moreover, 2D materials exhibit an extensive diversity of electronic properties—ranging from semiconducting (e.g., MoS_2_, WS_2_) to semimetallic (e.g., TiS_2_, WTe_2_) and metallic (e.g., NbS_2_, VSe_2_, Ti_3_C_2_T_*z*_)—which can be further tuned by factors including layer number, defect engineering, crystallographic configuration, and surface chemistry.^29^ Such material characteristics govern the 2D material interaction with light and molecules. For instance, certain metallic and semimetallic 2D materials can support surface plasmons, which can confine electromagnetic fields from the mid-infrared to terahertz regimes.^30^ Semiconducting 2D materials can exhibit strong exitonic effects, thereby influencing the local dielectric environment.^31^ Such phenomena make 2D materials a promising tunable platform for surface-enhanced spectroscopies, where enhancement mechanisms involving both electromagnetic field concentration (due to plasmon excitation, edge effects, excitonic effects, or interflake coupling) and chemical interactions (such as charge transfer between the analyte and 2D material) have been reported.^32–39^

In this work, we investigate the potential of using various liquid-phase-exfoliated (LPE) 2D nanomaterials as readily prepared platforms for enhancing molecular VCD signals. We selected a suite of 2D materials with diverse electronic properties for comparison (see Figure 1a-f): semiconducting 2H-WS_2_, semimetallic 2H-TiS_2_ and 2H-NbS_2_, metallic 3R-NbS_2_ and Ti_3_C_2_T_*z*_, and magnetic metallic 2H-VS_2_. As a model chiral analyte, we employed (R)- and (S)-1,1’-bi­(2-naphthol) (BINOL), a well-characterized molecule with distinct axial chirality and known VCD spectra.^3^ Composite thin films were fabricated by simply co-sonicating the bulk 2D material precursors directly with BINOL enantiomers in acetonitrile (ACN), followed by drop-casting the resulting dispersion onto double-side polished CaF_2_ substrates (Figure 1g). This approach explores whether intimate surface-to-volume ratio contact facilitated during the exfoliation process promotes impactful molecule-nanosheet interactions capable of amplifying VCD responses. We analyzed the resulting FTIR and VCD spectra to assess the degree of enhancement and spectral modifications induced by the different 2D materials, comparing the results to BINOL alone and BINOL on a simple gold thin-film platform. Our findings reveal material-dependent VCD enhancement, most notably with Ti_3_C_2_T_*z*_, and intriguing spectral changes, including apparent signal inversions, suggesting that LPE-derived 2D materials offer a versatile and accessible platform for modulating and enhancing molecular VCD signals.

**Figure 1:**
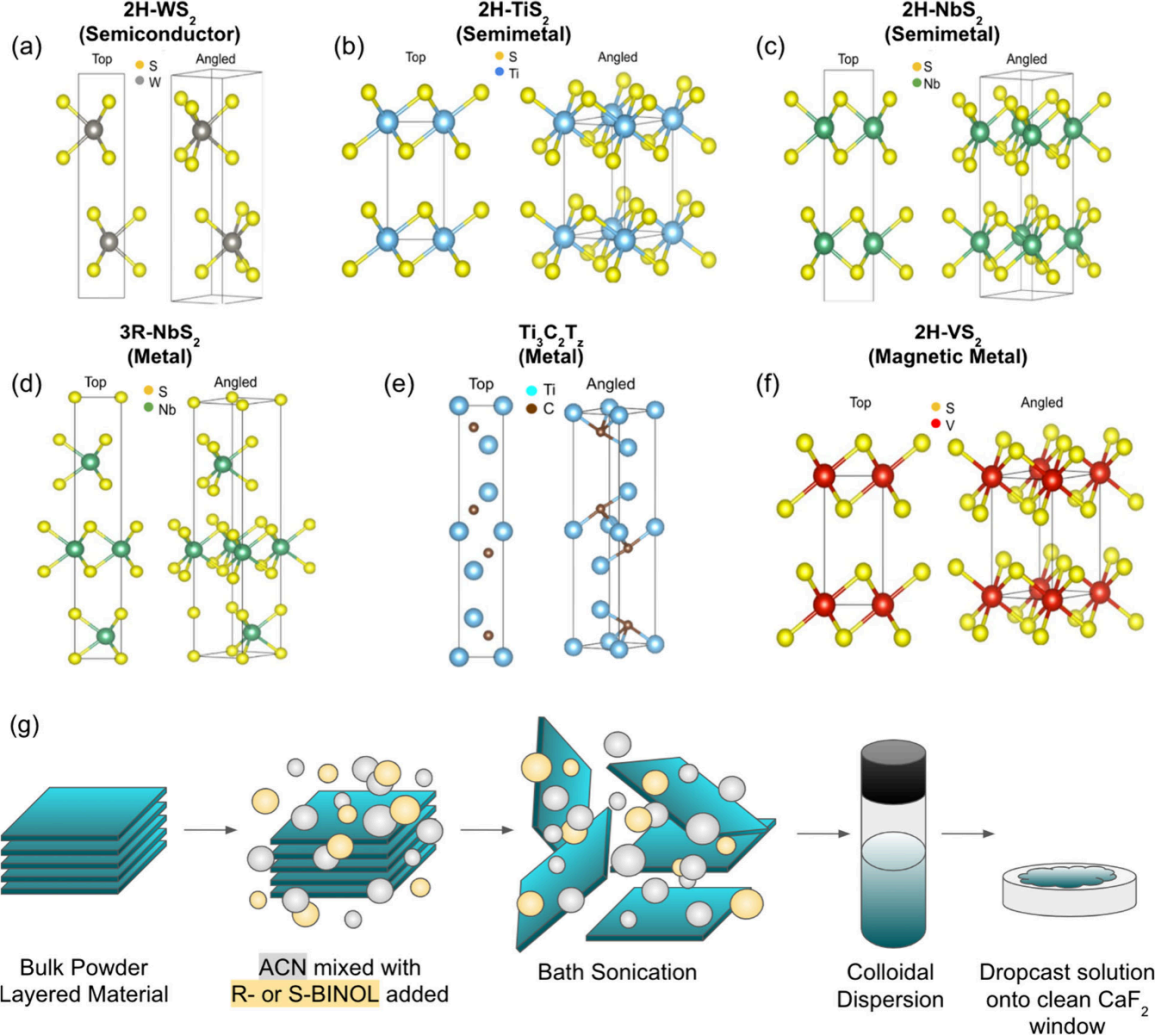
Crystallographic structures of selected 2D materials used to make thin-film composites for FTIR and VCD measurements: (a) semiconductor 2H-WS_2_, (b) semimetallic 2H-TiS_2_, (c) semimetallic 2H-NbS_2_, (d) metallic 3R-NbS_2_, (e) metallic Ti_3_C_2_T_Z_, and (f) magnetic metallic 2H-VS_2_. (g) Schematic showing the exfoliation process of 2D materials with R- and S-BINOL making a thin film on CaF_2_.

To assess LPE 2D nanomaterials as a platform for molecular VCD enhancement, composite films were prepared on double-sided polished CaF_2_ substrates (see Figure 1g and the Supporting Information for more details). The corresponding optical and chiroptical characteristics of the materials were evaluated using FTIR and VCD spectroscopy, respectively.

## FTIR Spectral Analysis

Figure 2 presents the FTIR absorption spectra (1650–1100 cm^–1^) for the six BINOL-2D material composites, alongside select control samples. The bare, uncoated CaF_2_ substrates show negligible absorption in this spectral region (apart from a baseline slope at lower wavenumbers). Control samples of LPE 2D materials drop-cast without BINOL exhibited minimal spectral features, confirming that the materials themselves do not possess significant intrinsic IR absorption in this fingerprint region. The spectra of R- and S-BINOL drop-cast directly onto CaF_2_ show the characteristic vibrational modes of the molecules, displaying identical, overlapping FTIR spectra as expected for these enantiomers. Key vibrational bands, labeled ‘i’ through ‘vii’, correspond to known BINOL vibrational modes determined using ComputeVOA,^40^ CompareVOA,^41^ Gaussian,^42^ and EnergyLog^43^ databases: ‘i’ ∼ 1620 cm^–1^ (naphthyl ring C=C stretch, -H rocking); ‘ii’ ∼ 1508 cm^–1^ (ring C=C stretch); ‘iii’ ∼ 1465 cm^–1^ and ‘iv’ ∼ 1430 cm^–1^ (antisymmetric ring stretching, -H rocking, −OH rocking); ‘v’ ∼ 1275 cm^–1^ (symmetric ring stretching, -H scissoring); ‘vi’ ∼ 1215 cm^–1^ (-H scissoring/rocking); and ‘vii’ ∼ 1145 cm^–1^ (antisymmetric ring stretch, -H scissoring including −OH group). Mode designations are also provided in Table 1 for ease of reference. A control sample with BINOL drop-cast onto a Cr/Au thin film on CaF_2_, representing a simplistic SEIRA thin-film reference,^16^ shows noisy BINOL FTIR features with potentially minor intensity variations compared to BINOL on bare uncoated CaF_2_ (see Figure S1 for FTIR and Figure S2 for VCD spectra).

**Figure 2:**
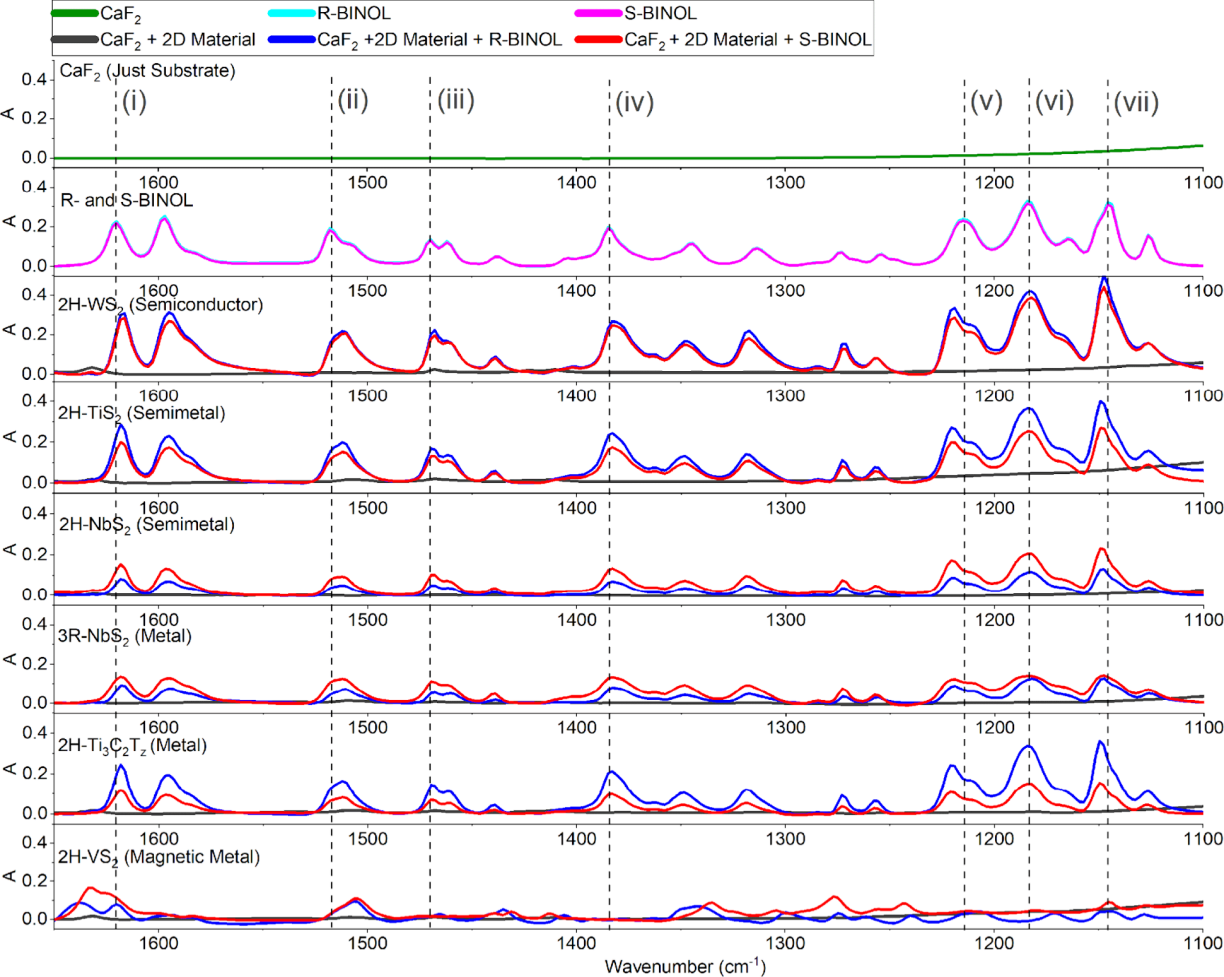
FTIR spectra of 2D nanomaterials mixed with R- and S-BINOL drop-cast onto CaF_2_ substrates and measured on the VCD spectrometer. The spectra represent 2D nanomaterial films with and without BINOL. The inset Roman numeral labels identify vibrational modes, as described in Table 1.

**Table 1:**
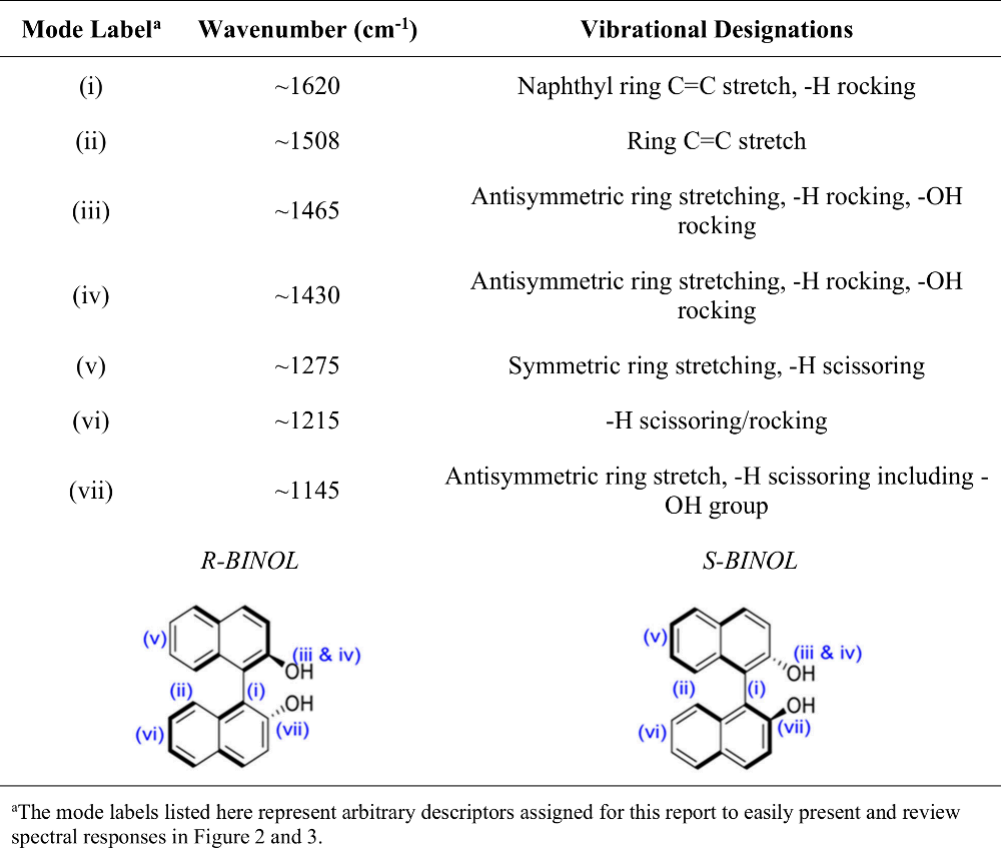
Vibrational Mode Designations for R- and S-BINOL Calculated Using ComputeVOA,^40^ CompareVOA,^41^ Gaussian,^42^ and EnergyLog^43^ Online Databases

Upon co-sonication and deposition with the BINOL-2D material dispersion on CaF_2_, the characteristic vibrational modes of BINOL clearly appear in the spectra, indicating successful incorporation of the analyte. However, distinct modifications of the vibrational bands relative to the BINOL-only control spectra are observed, varying significantly depending on the specific 2D material used. While most BINOL peaks remain largely aligned at the expected spectral positions across the different samples, notable shifts occur. For instance, mode ’i’ (∼1620 cm^–1^) appears slightly blue-shifted for the 2H-VS_2_ composite, while mode ’ii’ (∼1508 cm^–1^) is slightly red-shifted for the same material (see Figure 2). Such shifts likely represent perturbations of the molecular vibrational potentials due to interactions (e.g., physisorption or π-stacking) with the specific 2D material surface.

The FTIR intensities are also modulated by the various 2D materials. Relative to the BINOL-only control (∼0.2 absorbance units for mode ‘i’), the intensity of mode ‘i’ is slightly enhanced for metallic Ti_3_C_2_T_*z*_, semimetallic 2H-TiS_2_, and semiconductor 2H-WS_2_, while it is slightly dampened for magnetic metallic 2H-VS_2_, metallic 3R-NbS_2_, and semimetallic 2H-NbS_2_. Damping is more pronounced for modes ‘v’, ‘vi’, and ‘vii’ in the presence of 2H-VS_2_, 3R-NbS_2_, and 2H-NbS_2_, where the peaks become significantly weaker or nearly flatline. This suggests a SEIRA-like effect, which can involve both enhancement and damping depending on factors such as molecule–surface distance, orientation, and the dielectric properties of the substrate.^12,15,44,45^ Interestingly, differential intensity effects between enantiomers are observed in the IR spectra for some 2D materials: R-BINOL shows stronger absorption than S-BINOL for metallic Ti_3_C_2_T_*z*_ and semimetallic 2H-TiS_2_, whereas S-BINOL shows stronger absorption for metallic 3R-NbS_2_ and semimetallic 2H-NbS_2_ (particularly at mode ‘i’). This unexpected enantiomeric distinction in FTIR absorption intensity may indicate enantioselective adsorption geometries or interactions^46^ and thus warrants further investigation.

Significant peak broadening is observed for modes ‘ii’, ‘iii’, and ‘iv’ across most 2D material composites compared to the BINOL-only control. These modes involve vibrations of the aromatic rings and −OH groups, moieties likely to be involved in surface interactions. Such broadening often indicates a distribution of local environments or interaction strengths for the adsorbed molecules. For 2H-VS_2_, modes ‘iii’ and ‘iv’ are almost entirely absent, perhaps due to strong quenching interactions or altered vibrational selection rules upon adsorption. Furthermore, mode ‘v’ (∼1275 cm^–1^) appears to split into a doublet or develop pronounced shoulders for metallic Ti_3_C_2_T_*z*_, semimetallic 2H-TiS_2_, and semiconductor 2H-WS_2_ composites, suggesting lifting of conformer degeneracy or the emergence of distinct adsorbate populations.

It is worth noting that discernible Fano resonances were not observed in our work, unlike previous work from Zhou et al.^47^ who observed coherent coupling between vibronic states of the ligands and MXene bands, resulting in Fano resonances. The hybrid structure was formed through actual covalent bonding (achieved by deprotonation of the amine ligands) and may significantly contribute to the Fano effect. The lack of Fano resonances in the MXene IR spectra of our work suggests a weak coupling between the vibrational bands of BINOL and the MXene band of states. In our case, covalent bonding is unlikely since the BINOL −OH was not deprotonated. Hydrogen bonding between BINOL −OH and MXene −OH is likely to be the most prevalent chemical interaction, in addition to physisorption.

## VCD Spectral Analysis

The VCD spectra reveal the chiroptical response of the BINOL-2D material composites (Figure 3). The blank CaF_2_ substrate and the 2D materials without BINOL show a zero VCD signal, as expected for an achiral material. The BINOL-only control spectra exhibit the characteristic VCD couplets for (R)- and (S)-BINOL, which are mirror images with opposite signs and weak intensities (ΔA scale of ±0.0003, consistent with typical molecular VCD magnitudes of 10^–4^ to 10^–5^).^1^ In comparison to the responses discussed below, representative BINOL on the Au/Cr SEIRA-like control film shows a noisy VCD spectrum with potentially some enhancement but lacks clear, well-defined features comparable to those of the BINOL-only spectrum within this measurement regime (see Figure S2).

**Figure 3:**
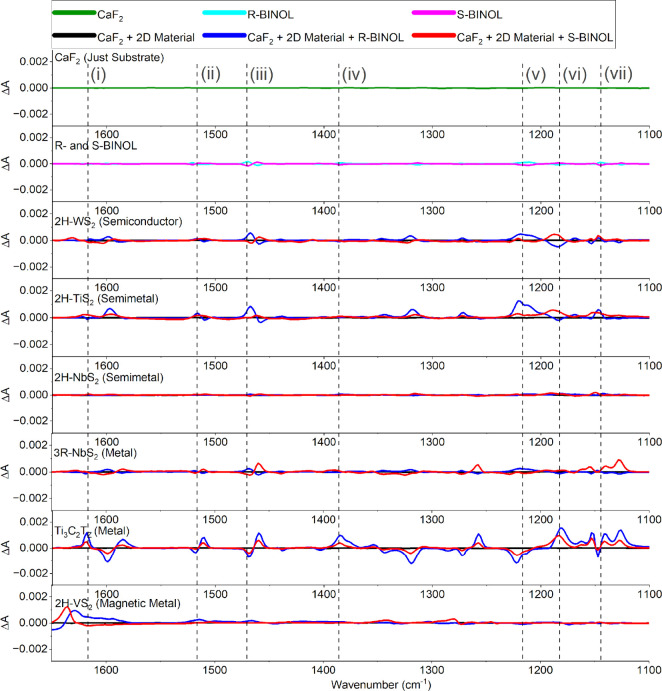
VCD spectra of 2D nanomaterials mixed with R- and S-BINOL drop-casted onto CaF_2_ substrates and measured in the VCD spectrometer. The spectra represent 2D nanomaterial films with and without BINOL. The inset roman numerals identify the vibrational modes, as described in Table 1.

In contrast, the introduction of LPE 2D materials leads to dramatic changes in the VCD spectra. The most striking observation is a significant enhancement of the VCD signal intensity upon interaction with several 2D materials, particularly metallic Ti_3_C_2_T_*z*_. Comparing the VCD intensity scale (ΔA ± 0.002) for the composites with the BINOL-only control (ΔA ± 0.0003), enhancements of 1 to 2 orders of magnitude (up to ∼100-fold for prominent peaks with Ti_3_C_2_T_*z*_) are evident. A close-up comparison can be viewed in Figure S3. This response is substantial for VCD spectroscopy and comparable to or exceeds enhancements reported for other systems like Co­(II)-doped gold clusters and is reminiscent of large enhancement factors observed in SERS with MXenes.^48^ The enhancement is broadband, affecting multiple vibrational modes across the measured range for metallic Ti_3_C_2_T_*z*_. Moderate enhancement is also suggested for semimetallic 2H-TiS_2_, while other materials like semiconductor 2H-WS_2_, semimetallic 2H-NbS_2_, metallic 3R-NbS_2_, and magnetic metallic 2H-VS_2_ show much weaker or negligible VCD signals under these conditions. We consider that only the high conductivity of metallic Ti_3_C_2_T_*z*_ providing the largest effect may point toward mechanisms linked to the electronic properties of the 2D material.

Beyond amplitude enhancement, intriguing changes in the BINOL VCD lineshapes and apparent handedness are observed. While enantiomers should yield mirror-image VCD spectra, the spectrum of R-BINOL on metallic Ti_3_C_2_T_*z*_ appears qualitatively similar in sign (though not identical in shape) to the spectrum of S-BINOL across several spectral regions (e.g., ∼1400–1500 cm^–1^ and ∼1100–1250 cm^–1^). A similar, though less pronounced, effect is observed for semimetallic 2H-TiS_2_ (see Figure S3). This inversion or flipping of the VCD handedness is unexpected. While VCD sign changes can occur due to conformational shifts or strong intermolecular coupling in solution or crystals,^49^ signal inversion induced by an achiral surface, as observed here, is not commonly reported and suggests complex underlying interfacial interactions. Potential explanations include (but are not limited to): (i) strong coupling between BINOL vibrational transitions and electronic states or resonances within the 2D material, significantly altering the rotatory strengths;^8,11–14^ ^,^ ^17,50,51^ (ii) adsorption-induced chirality, where the BINOL molecules impose a chiral footprint or arrangement on the interacting surface layer of the 2D material;^19,52^ or (iii) differential interactions leading to distinct preferred adsorption geometries or electronic states for R vs S enantiomers that possess different chiroptical signatures. Clear phenomenological confirmation remains unclear and requires further investigation.

The significant VCD enhancement observed (particularly for metallic Ti_3_C_2_T_*z*_) likely arises from a combination of mechanisms analogous to those invoked in SEIRA and SERS. An electromagnetic mechanism could involve enhancement of the local electric field experienced by the molecule due to the morphology (edges and defects) or electronic response (plasmons, excitons, and image dipoles) of the 2D nanosheets. While typical plasmon resonances for TMDCs/MXenes appear to be outside the mid-IR range as explored here, interband transitions or quadrupole plasmons identified in SERS may play a role.^35^ A chemical mechanism, likely involving charge transfer between molecular orbitals of BINOL and electronic bands of the 2D material, is also highly plausible, especially given the intimate analyte to 2D material spatial contact achieved during co-sonication and the known importance of charge transfer in SERS on Ti_3_C_2_T_*z*_.^39,53,54^ Charge transfer could modify the vibrational transition dipoles and rotatory strengths, leading to enhancement. Additionally, the surface terminations (T_z_, typically an amalgam of −O, −OH, −Cl, and −F terminations) on Ti_3_C_2_T_*z*_ could also play a critical role in mediating these interactions (e.g., potential BINOL to T_OH_ chemical or hydrogen bonding). The complex interplay between electromagnetic mechanism and chemical mechanism contributions (further complicated by the phenomena causing signal inversion) likely influences the material-dependent VCD responses observed here.

## 2D Material Electronic Property Comparison

Correlating the observed spectral responses directly with the broad electronic classifications (metallic, semimetallic, semiconducting, and magnetic) of the 2D materials reveals complex trends rather than simple relationships. As discussed, the most dramatic VCD enhancement and signal inversion effects were observed with metallic Ti_3_C_2_T_*z*_. The high electrical conductivity and density of states near the Fermi level for Ti_3_C_2_T_*z*_ are expected to support strong electromagnetic enhancement mechanisms (potentially involving surface interflake plasmonic resonances and/or intraflake localized field effects) and facilitate efficient charge-transfer interactions with adsorbed BINOL, consistent with theoretical and experimental SERS studies with MXenes referenced throughout. However, metallic 3R-NbS_2_ and semimetallic 2H-NbS_2_ exhibited negligible VCD enhancement. Rather, these materials induced significant FTIR signal dampening at lower wavenumbers. This divergence suggests that high conductivity alone is not a sufficient predictor for VCD enhancement, and factors such as the specific band structure relative to BINOL’s molecular orbitals, surface chemistry (including the T_*z*_ terminations unique to Ti_3_C_2_T_*z*_), and possibly nanoscale morphology or molecular adsorption conformation all play crucial roles toward enhanced molecular VCD behavior.

The semimetal 2H-TiS_2_ showed intermediate behavior, with moderate VCD enhancement and some indication of signal inversion, potentially reflecting its lower carrier density compared to Ti_3_C_2_T_*z*_ but higher than the semiconductor. The semiconductor 2H-WS_2_ produced only weak VCD signals but did cause some modulation of the FTIR spectrum (enhancement/splitting at certain modes) similar to that of metallic Ti_3_C_2_T_*z*_ and semimetallic 2H-TiS_2_. The presence of a band gap in 2H-WS_2_ might hinder efficient charge transfer as compared to metallic substrates but does not preclude electromagnetic effects or specific surface interactions influencing the molecular vibrational modes. Perhaps most distinct was the behavior of the magnetic metal 2H-VS_2_, which yielded weak VCD signals but caused significant FTIR peak shifts and dampening/disappearance of certain modes. The unique magnetic ordering in 2H-VS_2_ could introduce additional interfacial interaction pathways with BINOL or modify the surface electronic structure and adsorption behavior in ways distinct from nonmagnetic metallic TMDC analogs.

Overall, while the notable performance of metallic Ti_3_C_2_T_*z*_ aligns with expectations for surface-enhancement phenomena reliant on free carriers, the diverse responses across the full suite of materials indicate that a simple classification as metal, semimetal, or semiconductor is insufficient to predict nuanced FTIR and VCD spectral outcomes. A complex interplay involving specific electronic band alignment, surface functional groups, defect states, morphology, and potential magnetism may influence the molecule-nanomaterial interactions, resulting in the spectroscopic signatures observed here.

This work demonstrates the application of LPE 2D nanomaterials as a simple yet effective platform for enhancing the VCD signals of chiral molecules. By employing a straightforward co-sonication method to mix the model analyte BINOL with various 2D materials (WS_2_, TiS_2_, NbS_2_, Ti_3_C_2_T_*z*_, VS_2_) during exfoliation, followed by drop-casting, we observed significant modulation of BINOL’s chiroptical response. Most notably, the metallic Ti_3_C_2_T_*z*_ platform induced a substantial VCD signal enhancement for BINOL, reaching up to 2 orders of magnitude compared to baseline BINOL on a CaF_2_ substrate. Overall, the observed enhancement and spectral modifications were highly dependent on the specific 2D material used, highlighting the importance of the 2D material’s compositional and electronic properties. Beyond amplitude enhancement, interactions of molecular analyte with the 2D materials induced complex changes in both FTIR and VCD spectra, including peak shifts, band broadening, intensity dampening, and an apparent inversion of VCD signal handedness.

While the precise mechanisms underlying these observations require further elucidation (especially the signal inversion), our results suggest complex interplay between the adsorbed chiral molecules and the 2D nanomaterials. Both electromagnetic enhancement effects (local field concentration) and chemical mechanisms (charge transfer and altered vibrational potentials due to adsorption), analogous to those in SEIRA/SERS, are likely contributing factors. The signal inversion phenomenon points toward strong molecule–surface coupling effects not observed in standard analyte-only VCD measurements.

## Supplementary Material



## Data Availability

The data that support this work are available from the corresponding author upon reasonable request.

## Notes

Materials, preparation of BINOL-2D material dispersions, thin film preparation, and additional FTIR/VCD plots (PDF)
