# Supplementary material for: Liquid-Phase Exfoliated 2D Nanomaterials for Enhanced Vibrational Circular Dichroism of Chiral Molecules
Source: Nano Lett. 2025 Sep 17;25(39):14363–70. doi: 10.1021/acs.nanolett.5c03583 (PMC12981014; doi:10.1021/acs.nanolett.5c03583)
Supplement: Supplementary file 1 [file nl5c03583_si_001.pdf]

Supporting Information

Liquid-Phase Exfoliated 2D Nanomaterials for Enhanced Vibrational Circular Dichroism of Chiral Molecules

Aria T. Ballance,[1] Urice Tohgha,[2,3] Amy Morren,[2,3] Michael A. Susner,[2] Jennifer S. Shumaker-Parry,[1] and Peter R. Stevenson[2]**

[1]Department of Chemistry, University of Utah, Salt Lake City, Utah 84112, USA

[2]Air Force Research Laboratory, Materials and Manufacturing Directorate, Wright-Patterson Air Force Base, Dayton, Ohio 45324, USA

[3]Azimuth Corporation, a Core4ce LLC, 2970 Presidential Drive. #200, Fairborn, Ohio 45342, USA

*Corresponding author: shumaker-parry@chem.utah.edu

*Corresponding author: peter.stevenson.2@us.af.mil

Materials. Bulk precursor powders of 2H-TiS_2, 2H-VS_2, 3R-NbS_2, 2H-NbS_2, and 2H-WS_2 (all >99% purity) were custom grown materials using chemical vapor transport growth methods outlined by Busch et al.[1] $Ti_3C_2T_z$ MXene precursor (Ti_3AlC_2 MAX phase) processing to prepare $Ti_3C_2T_z$ was synthesized via selective etching of Al as outlined Want et al.[2] (R)-(+)-1,1'-Bi(2-naphthol) (≥99%) and (S)-(−)-1,1'-Bi(2-naphthol) (≥99%) were purchased from Sigma Aldrich. Anhydrous acetonitrile (ACN, 99.8%). Calcium fluoride (CaF_2) windows (13 mm diameter x 1 mm thickness) were purchased from OptoCity. For the gold control sample, chromium (99.99%) and gold (99.99%) deposition materials were used.

Preparation of BINOL-2D Material Dispersions. Stock solutions of R-BINOL and S-BINOL were prepared by dissolving 4 mg of each enantiomer in 4 mL of anhydrous ACN (concentration ≈ 1 mg/mL or 3.5 mM). For each exfoliation experiment, approximately 2 mg of a specific bulk 2D precursor material was added to 2 mL of either the R-BINOL/ACN or S-BINOL/ACN stock solution in a glass vial. The mixture was briefly degassed using three cycles of purging the mixture with nitrogen followed by putting the materials under vacuum. Liquid-phase exfoliation and mixing were performed simultaneously using bath sonication (Elmasonic P, 37 kHz) for 2.5 hours. Sonication was done at 40 ºC which was employed to minimize solvent heating and potential degradation, although side reactions involving ACN under sonication cannot be entirely excluded. This co-sonication approach aimed to facilitate intimate contact between the BINOL molecules and the 2D material surfaces as the layers delaminate.

Thin Film Preparation. After sonication, the resulting colloidal dispersion was allowed to settle briefly for at least 30 min. A 100 μL aliquot of the supernatant dispersion was carefully drop-cast onto a clean CaF_2 window (for VCD/FTIR). The solvent was allowed to evaporate slowly in a controlled environment under a gentle stream of N_2 in a desiccator to form a thin film.

Control samples were prepared similarly by drop-casting the R-/S-BINOL/ACN stock solution onto a CaF_2 window pre-coated with Cr (2 nm adhesion layer) and Au (35 nm) via electron beam evaporation (Au control). Samples of the 2D materials exfoliated in pure ACN (without BINOL) were also prepared and drop-cast onto CaF_2 substrates as further controls.

FTIR/VCD. Vibrational Circular Dichroism (VCD) and Fourier Transform Infrared (FTIR) absorption spectra were acquired simultaneously using a BioTools ChiralIR-2X FT-VCD spectrometer equipped with a photoelastic modulator (PEM) operating at 36.958 kHz for PEM1 and a liquid nitrogen-cooled mercury cadmium telluride (MCT) detector. The drop-cast film on the CaF_2 window was mounted in a BioTools SyncroCell rotating sample holder that was used to minimize linear birefringence artifacts. Spectra were collected over the range 2000–1000 cm^{-1} and plotted to show 1650-1100 cm^{-1} at a resolution of 4 cm^{-1}. For each sample, data were typically accumulated over 12 blocks, with 3120 scans per block, resulting in a total measurement time of approximately 12 hours to achieve adequate signal-to-noise for the weak VCD signals. Raw interferograms were processed using Grams AI software. Background correction was performed using Grams AI and Origin. VCD spectra are reported as differential absorbance, $\Delta A = A_L - A_R$ as described in the main text. ComputeVOA, CompareVOA, and Gaussian were used to generate approximations of the vibrational modes for R- and S-BINOL and compared with liquid-cell measurements acquired with 0.3M R/S-BINOL in deuterated chloroform.

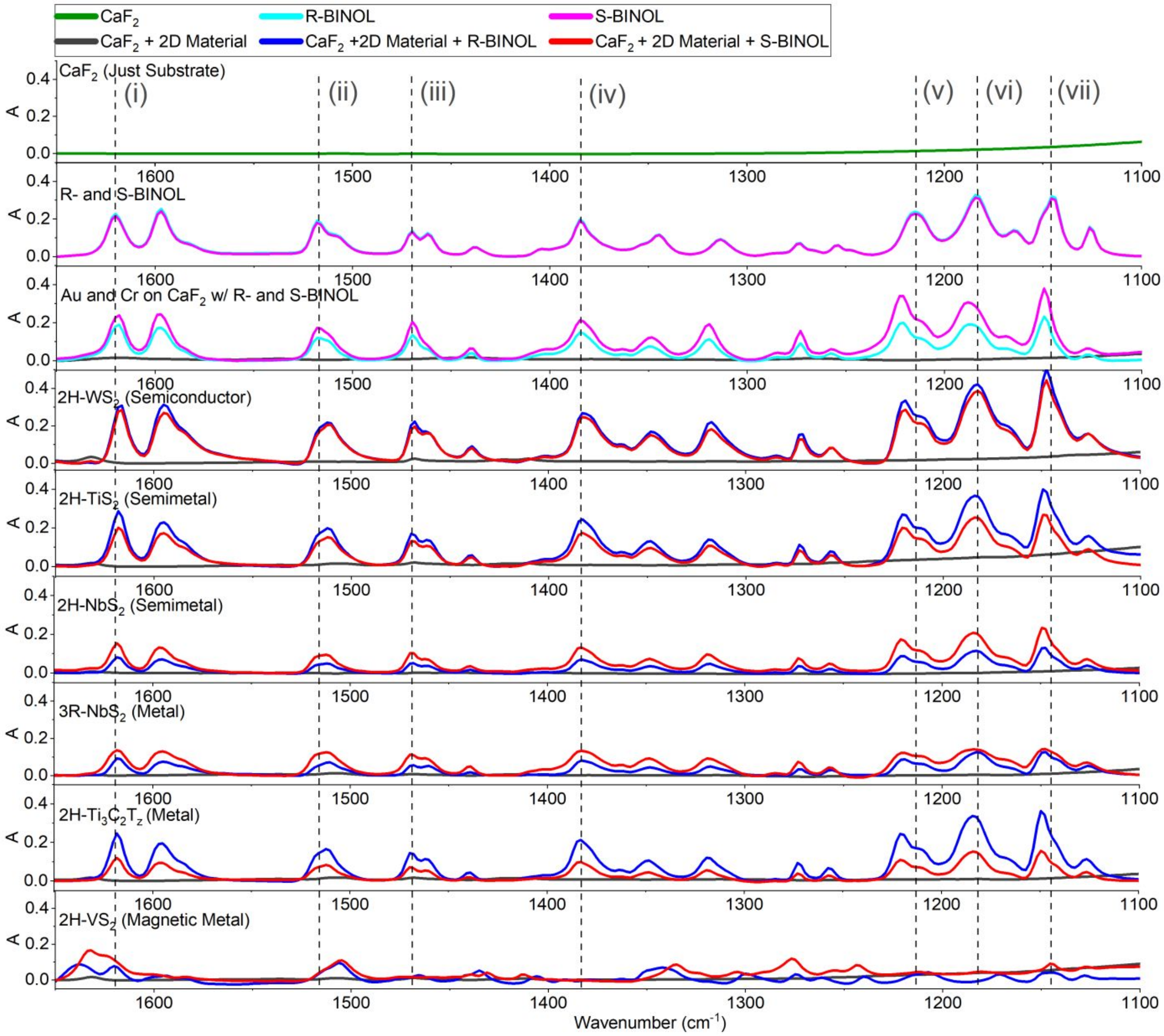

Figure S1. FTIR spectra of 2D nanomaterials mixed with R- and S-BINOL drop-casted onto a CaF_2 slide and measured in the VCD spectrometer. The spectra represent 2D nanomaterial films with and without BINOL. The inset roman numerals identify the vibrational modes as described in Table 1. This dataset also includes the control of Au and Cr on CaF_2 with and without R- and S-BINOL.

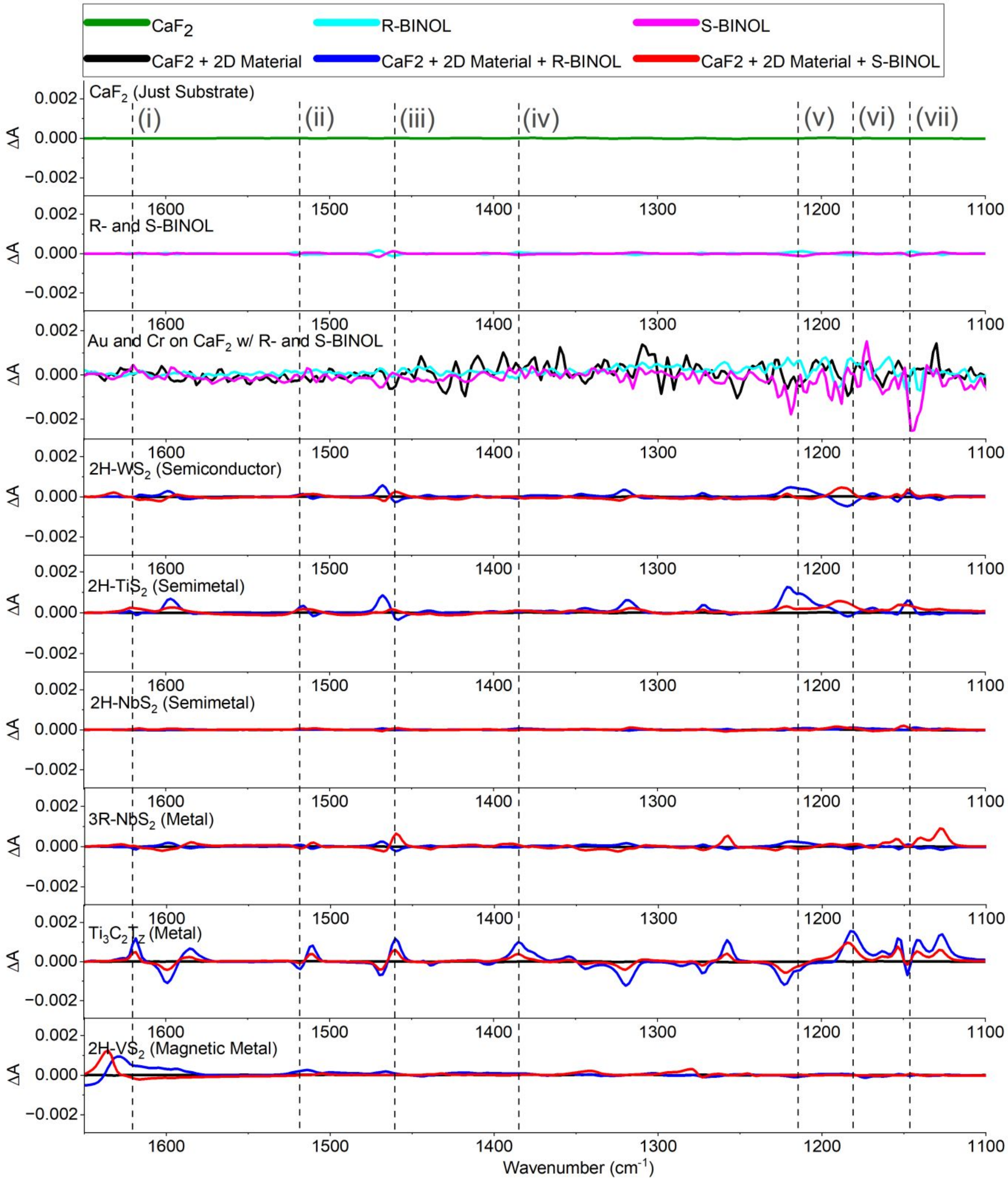

Figure S2. VCD spectra of 2D nanomaterials mixed with R- and S-BINOL drop-casted onto a CaF_2 substrate and measured in the VCD spectrometer. The spectra represent 2D nanomaterial films with and without BINOL. The inset roman numerals identify the vibrational modes as described in Table 1. This dataset also includes the control of Au and Cr on CaF_2 with and without R- and S-BINOL.

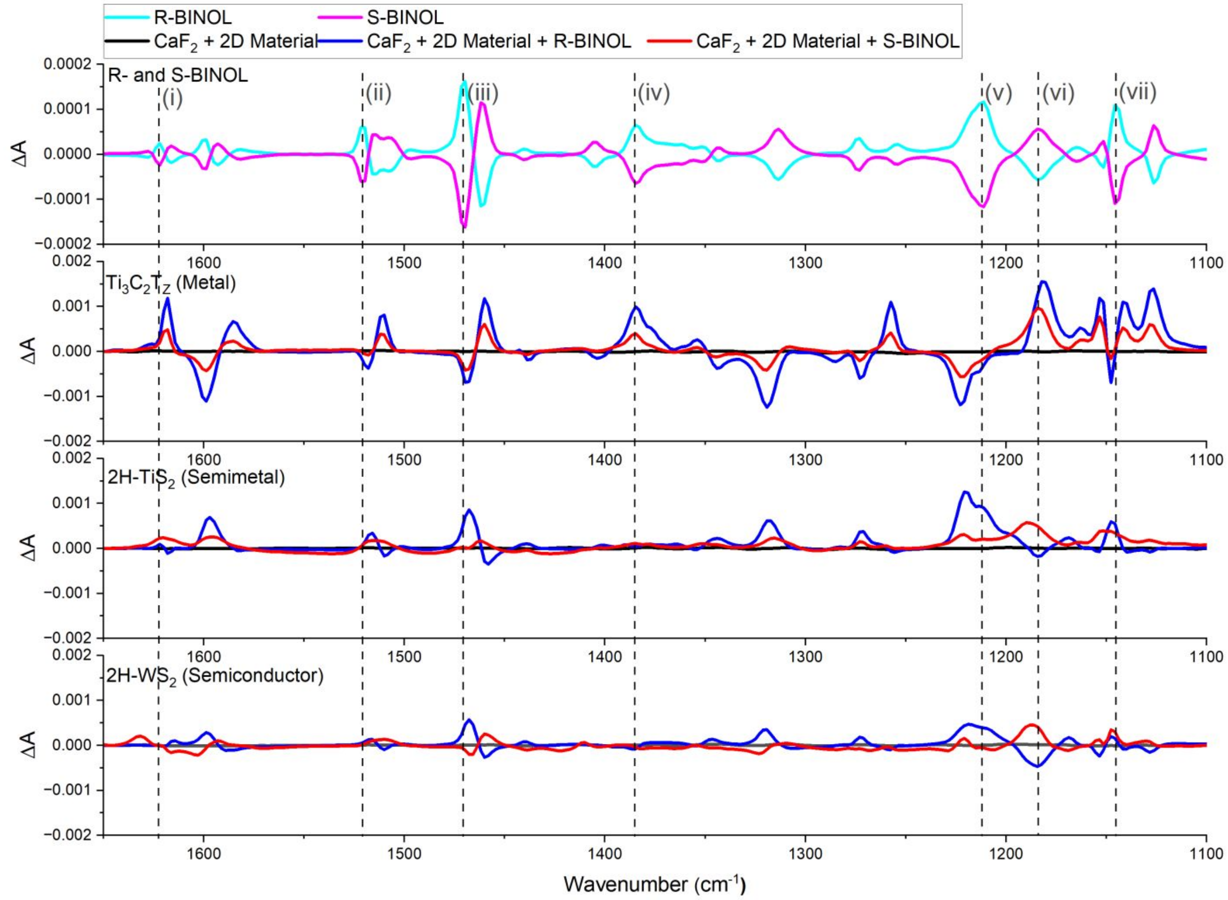

Figure S3. Zoomed in VCD spectra of R- and S-BINOL on CaF_2, $Ti_3C_2T_z$ with and without BINOL, 2H-TiS_2 with and without BINOL, and 2H-WS_2 with and without BINOL.

References

(1) Busch, R.T.; Sun, L.; Austin, D.; Jiang, J.; Miesle, P.; Susner, M.A.; Conner, B.S.; Jawaid, A.; Becks, S.T.; Mahalingam, K.; Velez, M.A.; Torsi, R.; Robinson, J.A.; Rao, R.; Glavin, N.R.; Vaia, R.A.; Pachter, R.; Kennedy, W.J.; Vernon, J.P.; Stevenson, P.R. Exfoliation Procedure-Dependent Optical Properties of Solution Deposited MoS_2 Films. *npj 2D Mater Appl.* **2023**, *7* (12).

(2) Wang, H.-W. N., M.; Page, K.; Wesolowski, D.J.; Gogotsi, Y. Resolving the Structure of Ti3C2Tx MXenes through Multilevel Structural Modeling of the Atomic Pair Distribution Function. *Chem. Mater.* **2016**, *28*, 349−359.
